# Understanding the Needs and Wishes of Older Adults in Interprofessional Treatment for Malnutrition and Sarcopenia: A Grounded Theory Study

**DOI:** 10.2147/JMDH.S507567

**Published:** 2025-03-11

**Authors:** Sandra D Boxum, Sabien H Van Exter, Jan-Jaap Reinders, Hans Drenth, Manon G A Van den Berg, Michael Tieland, Anjo Geluk-Bleumink, Sophie L W Spoorenberg, Evelyn Finnema, Philip J Van der Wees, Niek Koenders, Harriët Jager-Wittenaar

**Affiliations:** 1 Research Group Healthy Ageing, Allied Health Care and Nursing, Hanze University of Applied Sciences, Groningen, the Netherlands; 2 Science Department IQ Health, Radboud university medical center, Nijmegen, the Netherlands; 3 Department of Gastroenterology and Hepatology, Dietetics, Radboud university medical center, Nijmegen, the Netherlands; 4 Center for Dentistry and Dental Hygiene, University Medical Center Groningen, University of Groningen, Groningen, the Netherlands; 5 Research Group Interprofessional Education (IPE), Lifelong Learning, Education and Assessment Research Network (LEARN), Research Institute SHARE, University Medical Center Groningen, University of Groningen, Groningen, the Netherlands; 6 ZuidOostZorg, Organization for Elderly Care, Drachten, the Netherlands; 7 Department of Primary and Long-Term Care, University of Groningen, University Medical Center Groningen, Groningen, the Netherlands; 8 Center of Expertise Urban Vitality, Faculty of Sports and Nutrition, Amsterdam University of Applied Sciences, Amsterdam, the Netherlands; 9 School of Exercise and Nutrition Sciences, Institute for Physical Activity and Nutrition, Deakin University, Geelong, Victoria, Australia; 10 Denktank 60+ Noord, Steenwijk, the Netherlands; 11 Primary care group ‘Dokter Drenthe’, Assen, the Netherlands; 12 Health Science-Nursing Science and Education, University of Groningen, University Medical Center Groningen, Groningen, the Netherlands; 13 Research Group Living, Wellbeing and Care for Older People, NHL Stenden University of Applied Sciences, Leeuwarden, the Netherlands; 14 Research Group Nursing Diagnostics, Hanze University of Applied Sciences, Groningen, the Netherlands; 15 Department of Rehabilitation, Radboud university medical center, Nijmegen, the Netherlands; 16 Department of Physiotherapy and Human Anatomy, Faculty of Physical Education and Physiotherapy, Research Unit Experimental Anatomy, Vrije Universiteit Brussel, Brussels, Belgium

**Keywords:** malnutrition, sarcopenia, community-dwelling, interprofessional collaboration, person-centered care

## Abstract

**Background:**

Malnutrition and sarcopenia impact the physical health and quality of life of community-dwelling older adults. Managing these conditions requires integrating nutritional and exercise interventions delivered by professionals from diverse backgrounds. Interprofessional collaboration holds promise for providing integrated, person-centered care to older adults. However, to tailor such care, it is essential to understand the needs and wishes of older adults, which remain underexplored. This study aimed to understand the needs and wishes of community-dwelling older adults regarding interprofessional treatment for (risk of) malnutrition and sarcopenia.

**Methods:**

We conducted a grounded theory study. Data collection involved semi-structured interviews and focus groups with community-dwelling older adults who are undergoing treatment or have been treated for (risk of) malnutrition and/or sarcopenia. We systematically analyzed the data using open, axial, and selective coding and developed a conceptual model.

**Results:**

Interviews and focus groups were conducted with 18 older adults. Three selective codes were identified: 1) older adults need to be involved in their interprofessional treatment, 2) older adults need healthcare professionals to be well-informed about their interprofessional treatment, and 3) older adults need collaboration amongst involved healthcare professionals in interprofessional treatment. Our conceptual model addresses the needs and wishes of older adults in relation to interprofessional collaboration. Older adults’ needs highlight what is missing, while their wishes offer ways to fulfill these needs.

**Conclusion:**

Older adults’ need for involvement in interprofessional treatment can be met by engaging them actively in healthcare decisions and as partners to healthcare professionals. The need for well-informed healthcare professionals can be fulfilled by ensuring accessible healthcare information, the prevention of conflicting advice, and the prevention of repeating medical history. Finally, the need for collaboration among healthcare professionals can be fulfilled by healthcare professionals communicating openly and directly and working closely together.

## Introduction

With the global population aged 60 years and older projected to double from 2015 levels by 2050,^1^ the number of older adults at risk of or affected by malnutrition and sarcopenia is expected to increase substantially. Currently, the prevalence rates for malnutrition and sarcopenia among community-dwelling older adults are estimated at 7% and 13%, respectively.^2^,^3^ Malnutrition and sarcopenia are common among older adults and significantly impact physical health, independent functioning, overall quality of life, and mortality.^4–6^ Malnutrition results from inadequate nutrition intake or uptake, altering body composition and body cell mass.^7^ Sarcopenia is a muscle disease that may result from physical inactivity, inflammation, mitochondrial dysfunction, and malnutrition.^8^,^9^ Both conditions are characterized by reduced muscle mass and, in the case of sarcopenia, also loss of muscle strength.^8^,^10^ Effective treatment of these conditions requires a combination of nutritional and exercise interventions.^6^,^11^,^12^

Given the multifaceted nature of these conditions, multiple areas of expertise and skills, as well as a collaborative effort from diverse professionals across various disciplines, are needed to address the complex needs of older adults with, or at risk of, malnutrition and sarcopenia.^6^,^11^

Interprofessional collaboration (IPC) offers a promising approach to integrated, person-centered care for addressing (risk of) malnutrition and sarcopenia. IPC involves different health or social care professionals working together to provide health services and is characterized by shared accountability, interdependence between individuals, and clarity of roles and goals.^13^ The older adult is actively involved in the care process but is not a formal interprofessional team member. Unlike other forms of non-integrated collaboration in healthcare, in which professionals often work in parallel without coordinated efforts or shared objectives, IPC ensures a cohesive, team-based approach through joint treatment plans and a designated contact person to address the complex needs of older adults.^14^,^15^ So far, the perspectives of older adults remain underexplored, leaving it unknown how community-dwelling older adults view interprofessional treatment for (risk of) malnutrition and sarcopenia.

IPC centers around patient involvement, ensuring that older adults and their caregivers actively participate in healthcare. IPC can increase patient satisfaction and lead to better-informed patients.^16–19^ Poor patient involvement, in contrast, is linked to worse health outcomes.^20^ The World Health Organization (WHO) emphasizes that patient involvement improves patient safety in primary care and, therefore, must be encouraged.^21^ Understanding how older adults prefer to be engaged is essential for improving their healthcare outcomes, aligning with global healthcare initiatives, and enhancing person-centered care.^20^

Despite the benefits of IPC, research has predominantly focused on professionals’ perspectives, often neglecting the viewpoints of those who directly receive care. To our knowledge, no existing models link end-user needs and wishes with IPC. This oversight highlights the importance of qualitative studies to better understand the experiences and preferences of community-dwelling older adults with, or at risk of, malnutrition and/or sarcopenia. Understanding these perspectives is essential, as it can significantly impact the quality of person-centered care.^22^,^23^ Therefore, this study aimed to understand the needs and wishes of community-dwelling older adults regarding interprofessional treatment for (risk of) malnutrition and sarcopenia.

## Methods

### Study Design

We conducted a qualitative study using semi-structured interviews and focus groups. Grounded theory, as described by Strauss and Corbin, was used to construct theory from the data.^24^ This methodical, interpretive process was chosen to enhance understanding of older adults’ perspectives and generate theory informed by their experiences. We developed a conceptual model through iterative data analysis to show the interconnectedness of the data.^24^ By using both semi-structured interviews and focus groups, we captured in-depth individual insights and group dynamics, offering a comprehensive understanding of the older adults’ perspectives. We adhered to the 32-item Consolidated Criteria for Reporting Qualitative Studies (COREQ) checklist to ensure transparent and rigorous reporting of the study’s findings (Appendix 1).^25^

### Definition of “Need” and “Wish”

In this study, a “need” is defined as a felt state of deprivation that signals a fundamental requirement that cannot be repressed or replaced by another need.^26^,^27^ In contrast, a “wish” refers to a desire for a future outcome shaped by factors like value, expectancy, and practicality. A wish can emerge from anticipated consequences, outcomes, or actions.^26^,^28^,^29^ Unlike needs, wishes are flexible. As priorities change, wishes can be replaced by other wishes, reflecting aspirational goals rather than necessities.^26^ A wish represents the fulfillment of a need and can manifest in various forms, reflecting individual differences in desires and preferences.

### Eligibility Criteria and Recruitment

Participants were required to meet several criteria: being 65 years or older, living in the community and falling under the care of a general practitioner (GP), receiving treatment or having previously been treated for (risk of) malnutrition and/or sarcopenia, and being able to speak and read Dutch. Older adults with severe cognitive impairments that prevented them from making informed decisions were excluded. A qualified healthcare professional (HCP) evaluated potential participants, using their expertise to assess whether they met the inclusion criteria and could participate.

We used our direct and indirect professional networks and social media to reach out to professionals who work with older adults experiencing (or being at risk of) malnutrition or sarcopenia to assist in recruiting participants for the interviews and focus groups. Older adults who expressed interest were provided with informational leaflets and given the option to contact us via telephone or email. Alternatively, they could provide consent for their healthcare provider to share their contact details with the researchers.

We employed a purposive maximum variation sampling strategy to select participants for interviews and focus groups, ensuring diversity in gender, age, and the geographic living locations of the older adults. Recruitment spanned various regions in the Netherlands, covering the rural areas of Drenthe and Friesland and the cities of Nijmegen and Amsterdam. This approach facilitated a comprehensive understanding of diverse contexts across the country, where context refers to the various factors influencing participants’ experiences, including their geographical location (urban vs rural), social circumstances, and healthcare environment. These contexts shape how older adults interact with healthcare systems, experience conditions like malnutrition and sarcopenia, and perceive their treatment.^30^

Before participating in the interview or focus group, all participants gave written informed consent, including permission to publish anonymized quotes and participant characteristics.

### Data Collection

We conducted face-to-face interviews and focus groups between March and November 2023. We developed interview guides for the interviews and the focus groups (Appendix 2 and 3). Both guides were created using the I-change model.^31^ As preparation, we conducted a pilot interview with an older adult from the target group, and both guides were modified accordingly. For example, questions were rephrased to be more precise and more understandable for older adults, and some topics that did not generate in-depth responses were revised or expanded.

Interviews were held in the participants’ living environment or at their chosen location. The older adult was informed that an interview would last a maximum of one hour. One researcher, SB, conducted all interviews.

Focus groups were conducted in a designated meeting room, with each session scheduled for two hours, including time for arrival and post-discussion. One researcher, SB, moderated both focus groups. In the first focus group, researcher JJR co-moderated, and SvE was responsible for taking field notes as a documenter. For the second focus group, due to the smaller group size and our prior experience, only two researchers were involved, with SvE serving as both co-moderator and documenter. Following each session, SB, JJR, and SvE discussed field notes and made necessary adjustments to the interview guide. The guide for the final focus group session was adjusted based on the outcomes of the interviews and the first focus group (Appendix 4).

All interviews and focus groups were audio recorded, transcribed verbatim by a third party (Marinka^®^ Transcriberen), and subsequently analyzed using ATLAS.ti software (version 8.4, Scientific Software Development GmbH). Data collection ended after reaching theoretical saturation, at which point no new selective codes emerged.

### Reflexivity

The team’s diverse expertise enriched the exploration of the needs and wishes of older adults surrounding IPC. SB, drawing on expertise in physiotherapy, provided perspectives from primary healthcare practice. SvE, with a background in nutrition science, emphasized dietary considerations. JJR, as a work and organizational psychologist, offered insights into IPC. SB and SvE were PhD candidates throughout the study, and JJR held a postdoctoral position. SB had experience conducting interviews and was trained in qualitative research and focus group moderation. In addition to SB, JJR, and SvE, the research team encompassed individuals with diverse backgrounds in dietetics [MvdB, HJW], nursing [EF], nutrition and physical activity [MT], physiotherapy [NK, HD, PvdW], person-centered and integrated care [SdR], gerontology [HD], and qualitative research [NK]. Additionally, a representative of the older population [AGB] was involved, providing a comprehensive perspective on the analysis.

### Data Analysis

We performed a grounded theory analysis involving three steps: open, axial, and selective coding.^24^ SB and SvE independently read, re-read, and coded the transcripts using open codes. After this, they compared their open codes, engaging in open and constructive dialogue until consensus was reached. A constant comparative approach was employed to compare participant responses across interviews and focus groups. As a next step, axial codes were constructed. After all interviews and focus groups had finished, SB and SvE created selective codes to cluster the axial codes and identify connections in the data. Following the completion of the three stages of analysis by SB and SvE, all codes were reviewed within the research team to enhance mutual understanding and achieve consensus through four meetings with the entire research team. The first session focused on improving the open and axial codes. The second session was dedicated to discussing the axial and selective codes. The third and fourth sessions were devoted to constructing the conceptual model. The final axial and selective codes and the conceptual model were refined throughout the reporting process and subsequent manuscript drafts.

### Trustworthiness

ATLAS.ti software (version 8.4, Scientific Software Development GmbH) facilitated data aggregation and analysis, promoting transparency in code descriptions through participant quotes. The constant comparison method strengthened the study’s credibility by continuously comparing data and codes throughout the analysis. This method ensured that interpretations were grounded in the data and allowed for the refinement of axial codes. The ongoing improvement of the interview guides exemplified researchers’ commitment to reflect on the research methods and findings throughout the research process. Analytical memos were documented throughout the data analysis to record decisions and interpretations. This enabled the research team to confirm the study’s findings independently of the first authors’ biases or influences, further enhancing the confirmability of the findings.

## Results

Eleven interviews were conducted in Amsterdam and Drenthe, and two focus groups with seven participants were held in Nijmegen and Friesland. In Nijmegen, two older adults who had agreed to participate previously could not join the focus group at the location due to health issues. In Friesland, one older adult initially agreed to participate in a focus group but ultimately decided not to join, as he did not wish to participate in a group setting with multiple people. In Amsterdam, two other older adults preferred one-on-one interviews over group settings, so individual interviews were conducted instead of a focus group.

In one of the focus groups, a participant’s partner attended, and in another, a family member was present. The partner, actively involved in the participant’s care, contributed to the focus group discussion to provide support and add context to the participant’s experiences. The older adult in question requested this support, preferring to have assistance during the conversation. The partner provided informed consent, allowing us to use the input in the analysis. However, the family member just supported the other participant and did not provide data for analysis. Participant characteristics are detailed in Table 1.

**Table 1:** Participant Characteristics

	Participants (n)
**Interviews**	
Total of participants	11
Sex	
Female	8
Male	3
Age in years	
65–69	1
70–74	1
75–79	5
80–84	0
85–89	4
Region	
Drenthe	8
Amsterdam	3
**Focus groups**	
Total of participants	7
Sex	
Female	4
Male	3
Age in years	
75–79	1
80–84	2
85–89	4
Region	
Nijmegen	2
Friesland	5

The interviews lasted an average of 39 minutes, ranging from 23 to 56 minutes. The focus groups lasted 76 and 77 minutes. Theoretical saturation was reached after the second focus group session, marking the end of data collection.

Beyond the findings related to IPC, several nuanced experiences and needs of older adults emerged that go beyond the immediate focus of IPC. These insights are presented in a dedicated context paragraph, offering a more comprehensive view of older adults’ healthcare experiences. Following this broader context, seven axial codes and three selective codes were identified through analysis. These codes outline the needs and wishes of community-dwelling older adults regarding IPC in treating (risk of) malnutrition and sarcopenia. Finally, the conceptual model integrates these findings.

### Context

Older adults expressed a need for clear and understandable communication about their health and medical information. They preferred various communication channels to suit different situations and personal preferences, including telephone, in-person interactions, and digital platforms. Autonomy was highlighted as necessary, both in making healthcare decisions and in practical matters like transportation. Older adults emphasized the importance of autonomy and self-determination in managing their health. While they want access to comprehensive medical records and clearly explained care plans, they also expressed concerns about potential anxiety when reviewing these records.

Many older adults recognize the convenience of online access to their medical records, which helps them monitor aspects of their health, such as medication. However, despite these advantages, there remains a hesitation among older adults to engage with their primary care physicians online. Older adults highlight the ongoing need for in-person interactions with their HCPs.

### Selective and Axial Codes

#### Selective Code 1: Older Adults Need to Be Involved in Their Interprofessional Treatment

Older adults feel that they need to be actively involved in their treatment decisions and treated as equal conversation partners during discussions with their HCPs.

##### 1A: Older Adults Should Be Actively Involved in Their Healthcare Decisions

Older adults expressed a desire to actively take part in decision-making related to their healthcare, mainly when it involves treatment options and the selection of their care team. They highlighted the importance of shared decision-making, in which patients’ preferences and insights are considered when HCPs decide on their behalf.

Older adults value being consulted on treatment choices, providing input into which HCPs are on their care team, and discussing care planning and medication. While older adults appreciate this involvement, many prefer receiving clear summaries of formal multi- or interdisciplinary meetings rather than attending them directly. Ultimately, older adults want their preferences respected and have an active voice in decision-making while trusting HCPs’ guidance and expertise.

Participant (P3): They [general practitioner and physical therapist] have to. They shouldn’t just do anything without consulting me. I wouldn’t like that. I don’t want to be left out.

##### 1B: Older Adults Should Be Engaged as Partners to Their HCPs

Older adults emphasized that beyond decision-making, they want to be treated as equal partners in their healthcare, desiring mutual respect and engagement from their professionals.

They seek healthcare interactions where their opinions are heard, valued, and incorporated into care decisions, reinforcing a sense of equality in the relationship.

Older adults stressed the need for HCPs to listen to and acknowledge their unique perspectives. They felt that HCPs sometimes dominated the conversation, relying on their specialized knowledge and making older adults feel insignificant in decision-making. Older adults described situations where they felt treated as “just a number” rather than individuals with valid input. Participants expressed a need for collaborative, respectful conversations with their HCPs, where their voices are genuinely valued and their input plays a meaningful role in care decisions.

Participant (P1): For the patients, it is much better. Listen to the patients. Not like: I’m [a doctor] more educated, and you [the patient] are just a number. That’s how it goes. That doesn’t cross my mind. That is often the case these days. Not all doctors, but you have certain types who are so arrogant. They are so arrogant in healthcare. That bothers me.

#### Selective Code 2: Older Adults Need HCPs to Be Well-Informed About Their Interprofessional Treatment

Older adults need their HCPs to be well-informed, which can be achieved by making their medical information accessible to the professionals involved. Older adults hope this will prevent them from repeating their medical history to different HCPs. Accessible information would also diminish the conflicting advice they sometimes receive from HCPs.

##### 2A: Older Adults Wish for Their Medical Information to Be Accessible to Involved HCPs

Older adults expressed diverse perspectives on sharing their medical information. For some, the convenience of sharing treatment details between HCPs is paramount. They find it practical and reassuring when all involved professionals have access to their complete medical history, facilitating seamless care coordination and ensuring that new HCPs are informed from the start. They appreciate it when HCPs are well-informed about their treatments across different settings, indicating an efficient and well-coordinated healthcare system.

However, older adults have concerns about privacy and the extent of information sharing. Some older adults have specific preferences about what information should be shared and with whom. For instance, they may prefer that sensitive information, such as emotional well-being, is not shared without explicit consent. They also desire control over who can access their information, indicating that not all details should be available to every provider but only what is relevant to their care.

Participant (P4): At my age, what secrets do you have? I think it’s good when you become ill that they know what medications I’m on, what therapies I have.

##### 2B: Older Adults Should Not Have to Repeat Their Medical History to Different HCPs

Older adults expressed frustration with repeatedly recounting their medical history to different HCPs. As they often work with multiple specialists focused on various aspects of their care, they find it annoying and burdensome to share the same information repeatedly because of insufficient communication among HCPs.

Participant (P1): Because you have to tell them [dietitian and the geriatrics department] everything about what’s going on.

Partner of participant (P1): They are unaware of each other’s existence, so to speak.

##### 2C: Older Adults Should Not Receive Conflicting Advice From Their HCPs

Older adults reported receiving contradictory medical advice from different HCPs, which disrupts their treatment strategies and causes significant frustration. They specifically wish HCPs to refrain from offering advice outside their expertise to prevent inconsistencies with specialist recommendations. Older adults desire better coordination among HCPs to ensure that recommendations are aligned and accurate, avoiding unclear, false, or conflicting advice.

Participant (P9): She’s a nephrologist for the kidneys. She’s not for the muscles. So, I received advice that I thought wouldn’t work. And the physiotherapist also said, ‘Don’t bother with that; it’s not practical.’ I wasn’t happy with that advice [from the nephrologist] either; that’s not how it works.

#### Selective Code 3: Older Adults Need Collaboration Amongst Involved HCPs in Interprofessional Treatment

Older adults expressed a need for their HCPs to collaborate and communicate with each other. This is especially important when older adults are experiencing critical illness or are unable to care for themselves.

##### 3A: HCPs Should Work Closely Together

Older adults mentioned they find it comforting when HCPs collaborate as a team rather than in isolation, particularly during medical emergencies. Older adults emphasized the importance of improved agreement and cooperation between healthcare entities, such as pharmacists, general practitioners, and hospitals, regarding medication use. Insufficient communication in these areas has led to errors, highlighting the need for better coordination. Older adults appreciate HCPs working together seamlessly to ensure comprehensive and well-coordinated care, though they recognize that time constraints might limit this.

Participant (P2): It gives trust that there are HCPs who are not just here for themselves but are trying to work as a team. That doesn’t mean they have to contact each other every week; that’s nonsense, of course, because it’s not necessary at all. But, if necessary, I can say, ‘Get in touch with them,’ and they do. [.] It gives the most confidence because you are always dealing with adults who know the ins and outs and have a piece of background information you normally don’t give or can’t give.

##### 3B: HCPs Should Communicate Openly and Directly With Each Other

Older adults expressed that communication with their HCPs enhances their security and confidence. They desire seamless communication among professionals and want to be informed about these interactions without direct involvement. Effective communication is essential to them when HCPs have different opinions about their care. They also mentioned a greater need for inter-provider communication during serious illness.

Participant (P10): I like hearing that they [my general practitioner and dietitian] consult each other. My general practitioner, who was unsatisfied with something, consulted my dietitian; I thought that was very good. Because certain things are different for me than for someone else.

### Conceptual Model

**Figure 1:**
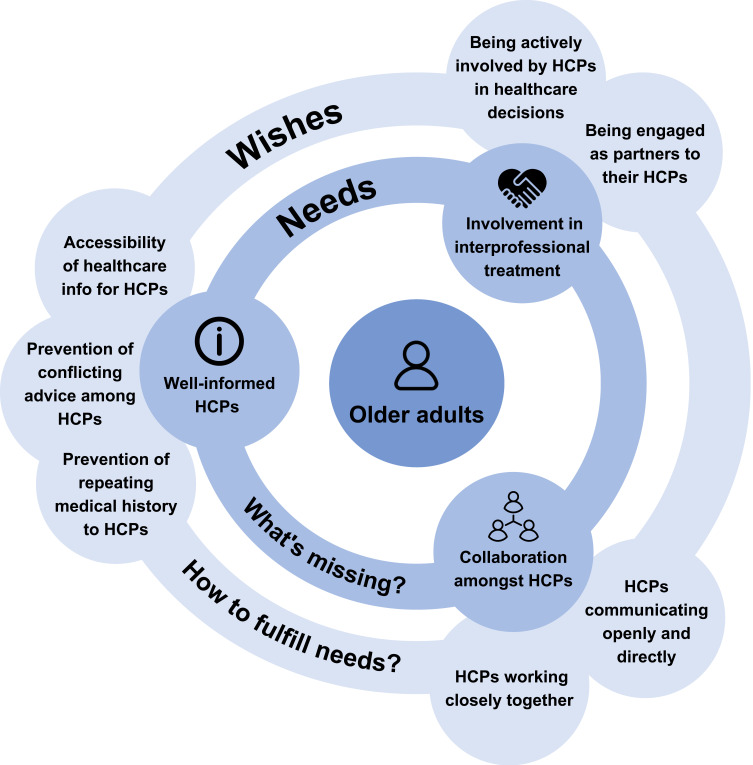
Conceptual model of interprofessional treatment of (risk of) malnutrition and sarcopenia: needs and wishes of community-dwelling older adults.

**Abbreviation**: HCPs, Healthcare professionals.

Our conceptual model visualizes the needs and wishes of community-dwelling older adults regarding interprofessional treatment for (risk of) malnutrition and sarcopenia (Figure 1). Older adults are at the model’s center, emphasizing their central role in a person-centered approach to care. The model consists of two layers: the inner circle represents needs, derived from the selective codes, and the outer circle represents wishes, derived from the axial codes. Needs define the fundamental requirements of older adults in the context of interprofessional treatment for malnutrition and sarcopenia, while wishes specify ways to fulfill these needs.

## Discussion

This study aimed to understand the needs and wishes of community-dwelling older adults regarding interprofessional treatment for (risk of) malnutrition and sarcopenia. Our conceptual model illustrates the needs and wishes of older adults with (risk of) malnutrition or sarcopenia. Older adults’ needs reflect their perception of what is missing in interprofessional treatment, while their wishes suggest how they believe these needs could be fulfilled. The older adults’ need to be involved in interprofessional treatment can be fulfilled by being actively involved in healthcare decisions and by being engaged as partners with their HCPs. The older adults’ need for well-informed HCPs can be fulfilled by making healthcare information accessible to HCPs, the prevention of conflicting advice among HCPs, and the prevention of repeating medical history to HCPs. Lastly, the older adults’ need for collaboration among HCPs can be fulfilled by HCPs communicating openly and directly and by HCPs working closely together.

Our study shows that older adults support data sharing among involved HCPs in their interprofessional treatment for malnutrition and sarcopenia. They appreciate having their complete medical history accessible to all HCPs, as it minimizes the need to recount their health information repeatedly and promotes consistency in treatment. However, older adults are also concerned about privacy. A previous study reported that older adults want to share health-related information but worry about losing control over their personal information and the risk of family members micromanaging when they can see all their medical information.^32^ Although acceptance of data sharing tends to increase as health needs grow, privacy concerns remain paramount, especially among more independent older adults.^32^

Our study aligns with the findings of a previous meta-ethnography, which shows that older adults consider strong interprofessional communication essential for integrated primary care.^33^ In that study, if older adults encountered conflicting advice or inadequate follow-up, they interpreted this as a breakdown in communication between HCPs. Older adults are particularly concerned about whether HCPs communicate openly with each other. Our study and the meta-ethnography show that older adults feel more secure and confident when HCPs maintain open and direct communication.

In our study, older adults expressed a desire for autonomy in their healthcare, emphasizing the need to be treated as partners in interprofessional treatment for malnutrition and sarcopenia. It is common for HCPs or family members to underestimate the older adult’s capacity to make autonomous decisions regarding their health, which can be detrimental to the feeling of self-control.^34^ The desire for participation aligns with the Self-Determination Theory (SDT) component of autonomy.^35^ Patients are more motivated to participate in decision-making when they feel they have control over their treatment choices and align with their values and preferences. Older adults in our study highlighted the need to have their preferences and values respected. By fostering a sense of agency and involving older adults as partners in their care, HCPs can enhance motivation and patient engagement.

Our study showed that older adults desire active decision-making. However, previous research specified that their involvement should focus on a “caring relationship”, “person-centered approach”, and “receiving information” rather than on “active participation in decision-making”.^36^ This shift may reflect evolving perspectives on healthcare autonomy between 2007 and 2024. According to the Attribution Theory, active involvement in healthcare can enhance older adults’ sense of control and self-esteem.^37^ When older adults are involved in decision-making and see positive results, they often feel that their efforts led to success, which boosts their self-esteem. However, when excluded from such decisions, they may attribute failures to external factors beyond their control, leading to frustration and disengagement.

The desire for active decision-making also aligns closely with the principles of shared decision-making (SDM), a component of person-centered care in which HCPs and patients work together to make healthcare choices.^38^ In SDM, HCPs share the best available evidence, presenting treatment options, risks, and benefits, while patients are encouraged to voice their values and preferences. Traditionally, SDM models have focused on a single patient-provider relationship. However, as healthcare becomes more complex and interprofessional, the concept of SDM has expanded to incorporate additional stakeholders, such as family members and other HCPs, shifting towards interprofessional shared decision-making (IP-SDM).^39^ Integrating IP-SDM could improve decision-making quality, strengthen patient-provider partnerships, and foster more coordinated, responsive care that aligns with patients’ comprehensive needs.

Older adults often experience multiple health conditions, with malnutrition and sarcopenia commonly appearing alongside other issues. Although we aimed to focus on these conditions, discussions frequently included care for other comorbidities. This overlap suggests that older adults perceive their health as interconnected rather than divided by individual conditions, reinforcing the importance of interprofessional treatment approaches for malnutrition and sarcopenia.

### Strengths and Limitations

A strength of this study is its use of a rigorously grounded theory methodology. Two researchers independently coded data, and a diverse group of experts interpreted the findings, enhancing the study’s trustworthiness. Researchers used the time between interviews and focus groups to analyze transcripts and refine the interview guide, allowing for deeper exploration of participants’ sentiments. Collaborative discussion sessions among all researchers contributed significantly to understanding older adults’ nuanced needs and wishes, which informed the thorough development of the conceptual model.

However, some limitations need to be acknowledged. Firstly, older adults in this study were unfamiliar with interprofessional care. Researchers had to explain the principle of interprofessional care during interviews and focus groups. While this ensured a basic understanding, more detailed insights might have been gathered if participants had prior knowledge or experience with IPC in their healthcare. Nonetheless, IPC for malnutrition and sarcopenia is rarely implemented in primary care, making it challenging to find suitable candidates with direct experience. Secondly, although the study’s minimum age requirement was 65 years, most participants were 75 years and older. This reflects the increasing prevalence of (risk of) malnutrition and sarcopenia with age, but the preferences of these older adults may differ from those of relatively younger participants. This demographic focus could limit the transferability of the findings to younger older adults.

### Implications for Clinical Practice and Future Research

The results of this study provide valuable insights into the needs of older adults in the interprofessional treatment of malnutrition and sarcopenia, which HCPs, policymakers, and researchers should consider.

For HCPs, these findings underscore the potential benefits of integrating older adults’ preferences into care practices through a structured interprofessional approach. Coordinated care that addresses interconnected health issues, such as malnutrition and sarcopenia, alongside other comorbidities, is essential. Interprofessional shared decision-making (IP-SDM) allows for unified treatment planning with the older adult and improves decision quality, reduces conflicting advice, and strengthens the partnership between patients and providers. This ensures older adults feel actively involved and respected in their care, leading to better health outcomes.

Policymakers should integrate older adults’ insights into policies that support person-centered and interprofessional care models. These policies should incorporate clear protocols for interprofessional communication, shared data access, and privacy protection. As privacy is a significant concern for older adults, policies should balance data sharing to facilitate seamless care with respect for patient autonomy and data control.

Additionally, researchers should build on these results by testing their applicability in practice and exploring further areas of IPC to improve care for older adults with (risk of) malnutrition and sarcopenia. Designing and implementing a structured interprofessional care pathway for these conditions could minimize the risk of conflicting advice and make it easier for older adults to navigate complex care networks. Moreover, interprofessional care pathways can improve treatment continuity and consistency, ensuring that older adults receive comprehensive, coordinated care tailored to their needs.

## Conclusion

This study contributes to a deeper understanding of the needs and wishes of community-dwelling older adults regarding interprofessional treatment for (risk of) malnutrition and sarcopenia. Older adults’ needs reflect their perception of what is missing in interprofessional treatment, while their wishes suggest how they believe these needs could be fulfilled. Older adults’ need for involvement in interprofessional treatment can be met by engaging older adults actively in healthcare decisions and as partners with healthcare professionals. The need for well-informed healthcare professionals can be fulfilled by ensuring accessible healthcare information, the prevention of conflicting advice, and the prevention of repeating medical history. Finally, the need for collaboration among healthcare professionals can be fulfilled by healthcare professionals communicating openly and directly and working closely together.

This study provides valuable insights for healthcare professionals, researchers, and policymakers to support person-centered interprofessional care for malnutrition and sarcopenia.
